# Effectiveness and safety of cataract surgery in laminar air flow device versus traditional scrubs: A 1-year non-inferiority pilot study

**DOI:** 10.3389/fmed.2023.987505

**Published:** 2023-02-23

**Authors:** Arnaud Artus, Filippo Fabro, Isabelle Cochereau, Georges Caputo, Ramin Tadayoni, Catherine Vignal, Olivier Galatoire, Flore Salviat, Damien Gatinel, Christophe Panthier

**Affiliations:** ^1^Department of Ophthalmology, Rothschild Foundation Hospital, Paris, France; ^2^Hôpital Lariboisière, Paris, France; ^3^RMD - Service de Recherche Clinique, Rothschild Foundation Hospital, Paris, France; ^4^Center of Expertise and Research in Optics for Clinicians (CEROC), Paris, France

**Keywords:** Surgicube^®^, cataract, surgery, endophthalmitis, ophthalmology

## Abstract

**Purpose:**

The study aimed to assess the safety and the non-inferiority of cataract surgery outside an operating room using the Surgicube^®^, a mobile laminar airflow (LAF) device.

**Settings:**

This single-center study was conducted at the Rothschild Foundation, Paris, France.

**Design:**

This is a retrospective cross-sectional study.

**Methods:**

All patients operated on for cataracts using the Surgicube^®^ between February 2020 and February 2021 were included and controlled by a cohort of patients operated on for cataracts in the traditional theater during the same period. Patients with a postoperative follow-up of less than 1 month were excluded. Data collection was carried out using the patient’s medical record. The primary endpoint was the evaluation of the number of endophthalmitis in the two groups. The secondary judgment criteria were the analysis of the various complications and the Logmar visual acuity at 1 month in the two groups. All the patients underwent an OCT retinal examination.

**Results:**

A total of 923 randomized patients who underwent cataract surgery between 2020 and 2021 have been included in the study. Among them, 448 patients were operated on using the Surgicube, and 475 patients underwent surgery in the traditional operating room using the same lens phacoemulsification technique. There are no significant differences between the two groups (*p* > 0.05).

**Conclusion:**

Cataract surgery using the Surgicube^®^ outside a conventional operating room seems non-inferior to conventional scrub.

## Introduction

**FIGURE 1:**
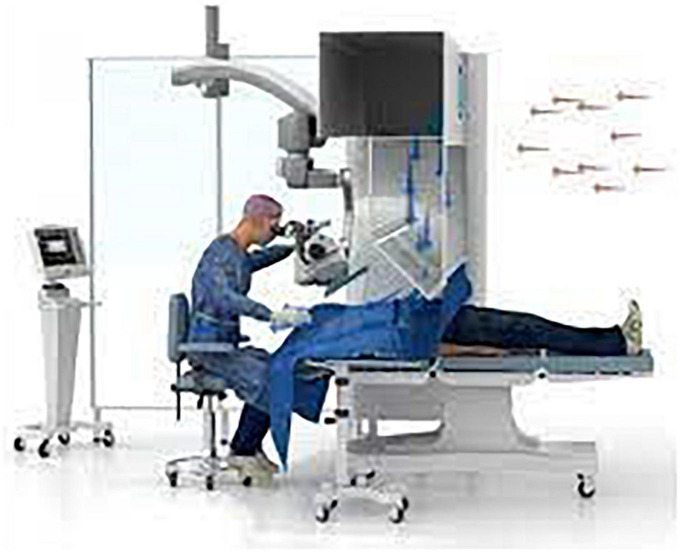
Surgicube: A mobile operation theater system.

Cataract surgery is one of the most performed surgeries in the world. Access to this surgery traditionally requires an operating room with air quality standards in France classified as International Organization for Standardization 7 (ISO 7) (1) to limit the incidence of infectious complications and mainly endophthalmitis. Faced with the growing demand for this surgery, the cost of building a traditional stationary theater leading to inaccessibility in certain rural areas or developing countries, mobile operating room systems for cataract surgery have been described (2, 3), as well as devices of “office-based cataract surgery” (4), allowing the operation of cataracts outside of a standard scrub. Mobile devices delivering a flow of sterile laminar air to the operating area with ISO 5 standards have now been used for a few years. Surgicube^®^ (Surgicube International BV, Hollande) is the one that has had CE marking since 2005 (Figure 1).

A recent study (5) measured the level of microparticles at the surgical site with and without a laminar air flow device (LAF) during cataract operations, showing a significant reduction during its use. Parker et al. (6) studied the feelings of patients operated with the Surgicube^®^ through 789 corneal transplants. Furino et al. (7) analyzed the ArcSterile^®^ (Arc Sterile, Spain), a mobile unit similar to the Surgicube^®^, on more than 10,000 intra-vitreal injections (IVT). All these studies did not report a higher preoperative or postoperative complication rate than the data known from the literature in a conventional theater. To the best of our knowledge, no study has investigated the results of cataract surgery outside of a theater with a mobile device having a LAF.

This study aims to evaluate the non-inferiority and safety of cataract surgery with the Surgicube^®^ by comparing it to a group of patients operated on standard theater.

## Patients and methods

### Patient selection

It was a retrospective, controlled, monocentric, multi-operator study carried out at the Rothschild Foundation Hospital in Paris concerning patients operated for cataract surgery with Surgicube^®^ versus a cohort of patients operated in a standard theater. The inclusion criteria for both groups were as follows: must be an adult and must be eligible for the pure topical protocol (no sedation and no other anesthesia except topical anesthesia). The patients were operated on between February 2020 and February 2021 with the Surgicube^®^ or in the conventional theater. We used a “date randomization.” Only patients planned with topical anesthesia only have been selected for this study. Each patient chooses the operating day. Following our general theater planning, some patients were admitted to the classic theater and some others to the Surgicube, according to the day they had chosen. Patients with a postoperative follow-up of less than 1 month were excluded. A total number of 93 patients has been excluded. Patient follow-up was carried out by ophthalmologists. For each patient, data were collected from the medical file of the Adolphe de Rothschild Foundation up to 1 month postoperatively. The data collected are as follows: visual acuity, ocular tone, and the presence and type of preoperative and postoperative complications (posterior capsular rupture with or without vitreous loss, intraoperative iris floppy syndrome or iris prolapse, iris or ciliary body injury, lens materials dropped into vitreous, suprachoroidal effusion with or without hemorrhage, transiently elevated intraocular pressure, cornea edema, toxic anterior segment syndrome, endophthalmitis, retained lens materials, hyphema, and Irvin–Gass syndrome).

### Surgical protocol

All patients were examined by their surgeon, with particular attention to the need for sedation according to the patient’s history and psychological profile. The indication for surgery was phacoemulsification with the placement of an implant in the capsular bag. Intracameral cefuroxime antibiotic prophylaxis was administered systematically in both groups. Postoperative treatment included a topical antibiotic (1 week), a non-steroidal anti-inflammatory (1 month), and cortisone antibiotic eye drops (1 month). Patients in the Surgicube^®^ group underwent topical anesthesia with tetracaine or oxybuprocaine, followed by additional intracameral lidocaine. They were prepared by the nurse and installed on a stretcher, with only the head arriving at the level of the sterile operating area by the laminar air flow. There was no anesthetist or peripheral venous line. The control group had topical anesthesia followed by an intracameral lidocaine injection. An anesthetist nurse was always present in the room. Patients had to change into an overall.

### Satisfaction survey

We asked the surgeons to fill out a satisfaction form rated from 1 to 10, their preference between operating with the Surgicube^®^ or conventional block, and we also recorded the average time of surgery, all for the 97 first interventions in the Surgicube^®^ group.

### Judgment criteria

The main judgment criterion is the evaluation of the number of endophthalmitis in the two groups. The secondary judgment criteria are the evaluation of the various complications, the visual acuity (VA) in the LogMar scale at 1 month, and the non-inferiority of the device.

### Statistical analyses

Qualitative and binary variables are expressed in number and percentage. The quantitative variables are in mean and standard deviation. The primary endpoint was calculated with Fischer’s test. The secondary judgment criteria are also calculated using Fischer’s test. All statistical analyses were performed using Statview^®^ software (SAS institute, Inc.) and Excel^®^ software (Microsoft, Corp., USA). A *P*-value of <0.05 was considered statistically significant. The threshold of non-inferiority corresponds to the greatest loss of efficacy compared to the reference treatment that can be consented to, taking into account the other advantages offered by the treatment.

## Results

A total of 448 eyes were included in the Surgicube^®^ group and 475 eyes in the control group. Patient baseline characteristics are presented in Table 1. The number of endophthalmitis was 0 in the Surgicube^®^ group and 1 (0.2%) in the control group; the difference between the two groups was non-significant (*p* > 0.05). The total number of complications in the Surgicube^®^ group was 36 (7.7%) and 43 (9%) in the control group; the difference between the two groups was non-significant (*p* > 0.05), and the detail of the complications is presented in Table 2. The number of posterior capsule ruptures was 0 in the Surgicube^®^ control group, six (1.26%) in the control group, odds ratio (OR) = 0.16, and confidence interval of 95% (IC) [0.004; 1.39] (*p* > 0.05). The postoperative LogMar visual acuity is 0.05 ± 0.15 and 0.006 ± 0.15, respectively, in the Surgicube^®^ group and the control (*p* > 0.05). There is no difference among the types of IOL that have been used. In the Surgicube^®^ group, 31 multifocal lenses and 68 toric lenses have been used compared to 32 multifocals and 72 torics in the control group. The mean duration of follow-up in months was 1.5 ± 1.2 for the Surgicube^®^ group and 1.4 ± 1.1 for the control group; this difference was not significant (*p* > 0.05). There is no more relative risk of being operated on in the Surgicube^®^, OR = 1. Concerning the surgery satisfaction form, 17 surgeons answer the questionnaires of the first 97 operations. The degree of satisfaction was 8.85/10. Only 23% of the surgeons would have preferred to operate in the conventional theater. The average operating time of these first interventions was 13.7 min [5.1; 29.7] in comparison to 15.2 min [4.9; 39.2] in the normal operating room (*p* > 0.05).

**TABLE 1:** Patient baseline characteristics, including age, laterality, sex, and preoperative visual acuity.

		LAF group *N* = 468	Control group *N* = 475
Age (years)	Average ± SD Range	70.0 ± 9.2 31–92	71.1 ± 9.2 50–88
**Eye**			
Right	*N* (%)	235 (50.2%)	241 (50.7%)
Left		233 (49.8%)	234 (49.3%)
**Sex**			
Male		228 (48.7%)	225 (47.3%)
Female		240 (51.3%)	250 (52.7%)
Preoperative visual acuity (LogMar)	Average ± SD Range	0.34 ± 0.14 0–1	0.33 ± 0.15 0–1

LAF, laminar air flow device; N, number.

**TABLE 2:** Perioperative and postoperative complications in two groups.

Complication	LAF group	Control group	OR [CI 95]	*p*
IOH, *n* (%)	21 (4.5%)	18 (3.8%)	0.7 [0.4: 1.4]	>0.05
Irvin Gass, *n* (%)	10 (2.1%)	10 (2.1%)	1 [0.4: 2.7]	>0.05
PCR, *n* (%)	0 (0%)	6 (1.3%)	0	>0.05
Endophthalmitis, *n* (%)	0 (0%)	1 (0.2%)	0	>0.05
TASS, *n* (%)	0 (0%)	1 (0.2%)	0	>0.05
Floppy iris, *n* (%)	2 (0.4%)	0 (0%)	∞	
Persistence crystalline mass, *n* (%)	1 (0.2%)	3 (0.6%)	0.3 [0: 3.3]	>0.05
Corneal edema, *n* (%)	1 (0.2%)	4 (0.8%)	0.25 [0: 2.25]	>0.05
Hypaema, *n* (%)	1 (0.2%)	0 (0%)	∞	
Total, *n* (%)	36 (7.7%)	43 (9%)	0.8 [0.5: 1.3]	>0.05

LAF, laminar air flow device; N, number; OR, odds ratio; CI 95, confidence interval at 95%; IOH, intra-ocular hypertension; PCR, posterior capsule rupture; TASS, toxic anterior segment syndrome.

## Discussion

This study on the incidence of endophthalmitis and the non-inferior safety of cataract surgery did not show any significant difference when it was delivered in a conventional block or a room with a mobile device having a sterile LAF.

For many years, ventilation with sterile LAF has been used in the operating room to prevent airborne contamination and infection rate, mainly in orthopedic surgery (8). Subsequently, mobile devices were developed to provide a sterile LAF directly in the operating area, reducing the presence of harmful pathogens in an operating room with a traditional ventilation system (9). Osher et al. (5) studied the l’Operio^®^ (ToulMeditech, Sweden), a mobile device with a horizontal LAF. They measured on 116 cataract operations the number of particles according to their size 0.5, 1, and 5 μm at different times in an operating room with and without a sterile LAF. They objectified a statistically significant decrease in the average number of particles of at least 79% in the operating area when the device was on. The second phase of their study concerned the analysis of the lint fibers found by the surgeon during the intervention by making the difference between those brought by an instrument and those found directly on the operating field on 99 cataract operations with LAF and 50 without LAF. Lint fibers were identified in 18% of eyes in the control group and 16% of eyes in the LAF group. The number of lint fibers carried into the sterile field was similar in each group, but the incidence of lint fibers falling onto the sterile field was significantly reduced from 6% (3/50) to 0% (0/99) when the LAF was used. There are very few clinical studies on the incidence of infectious complications with the use of a mobile system with LAF in ophthalmology. Furini et al. (7) analyzed more than 10.000 IVTs with the ArcSterile^®^, and they declared no endophthalmitis. The complications found were classic IVT’s complications, subconjunctival hemorrhages, and ocular hypertonia. A cataract is the leading cause of blindness and visual impairment worldwide and is a major public health issue (10). Cataract surgery is reproducible surgery, with a short operating time, rapid recovery, and few complications. Its demand throughout the world is growing, and conventional surgical infrastructures with an operating room are limited, especially in certain rural areas or developing countries. These mobile devices with sterile LAF can provide easier access to cataract surgery. Indeed, the manufacturing cost of a LAF is five to six times less expensive than a conventional operating room. It can also be moved from one place to another. Endophthalmitis remains a rare, 0.006%–0.04% (11), but dramatic complication of this surgery with often appalling visual prognosis. A meticulous procedure, as well as an intraoperative and postoperative antibiotic protocol, is used to reduce the incidence. Air quality in operating rooms is just as important in limiting the risk of airborne contamination. Air filtration guidelines for operating rooms have been determined by the American Society of Heating, Refrigerating, and Air-conditioning Engineers (ASHRAE 170) (12). The Surgicube^®^ is an innovative strategy for performing cataract surgery. It reduces the number of suspended particles in the air by a laminar downflow technique directly on the surgical field (13). Therefore, the surgical field may be considered aseptic and satisfies the requirements of ISO 5 quality level air classification, mandatory for an operating room (1). ISO 5 means air containing no more than 100 particles per cubic foot of air of a size at least 0.5 μm or larger in diameter (3,520 particles per cubic meter), which is better than ISO 7 [air containing no more than 10,000 particles per cubic foot of air of a size at least 0.5 μm or larger in diameter (352,000 particles per cubic meter)]. The narrowest Surgicube^®^ is 2.6 meters wide and has a sterile working area of 1.8 m. The widest Surgicube^®^ is 3.8 m wide and has a sterile working area of 2.2 ms wide. It is equipped with a real-time analyzer of the number of colony-forming unit (CFU) per m^3^ and these go into alarm if the number of CFU exceeds 0.45 m^3^ which remains, however, below the recommended standards despite the absence of a standard for CFU count measurement (12). Moreover, the Surgicube^®^ is a mobile device that can be positioned in any room that has the necessary space, with no restrictions or specific regulatory requirements. However, laminar airflow devices do not control other parameters of the operating environment such as humidity or room temperature, which can have an impact on the infection rate or the wellbeing of the surgical team and, therefore, on its performance.

To the best of our knowledge, this study is the first to analyze the infectious risks of cataract surgery outside a conventional operating room with a mobile device LAF by comparing it to a cohort of patients operated in a traditional theater with a conventional ventilation system. Our results are reassuring and suggest that cataract surgery with a Surgicube^®^ device could be non-inferiorly safe and effective. We had no endophthalmitis in the Surgicube^®^ group against one in the control group, with no significant difference between the two groups. The endophthalmitis of the control group could be explained by a complicated cataract with a posterior capsule rupture. We also note a significant difference in the number of posterior capsular ruptures in the control group 6 (1.26%) versus 0 in the Surgicube^®^ group. Thanks to the retrospective reading of the medical files, we can explain this difference because these complications were on cataracts identified as more difficult preoperatively.

Our study has, however, some limitations. It is a retrospective study with a systemic selection bias. We have the correct power, but the incidence of endophthalmitis being rare is not yet significant enough, and the results have to be confirmed by a prospective study on a larger cohort. It would also be interesting to study the patients’ feelings s using a satisfaction form of their surgery.

## Conclusion

In conclusion, our study shows that cataract surgery with a laminar air flow device, outside a conventional operating room, seems to be non-inferior to the traditional theater. This could lead to big changes in our daily practice in the years to come. A multicentric prospective randomized study with a larger sample is needed to confirm these results.

### What was known

• Mobile LAF devices are already well used in surgery, particularly in orthopedic surgery, and provide additional security against airborne contamination in the operating room.

### What this paper adds

• This is the first study to investigate the safety and the non-inferiority of a mobile LAF device for cataract surgery, and the results appear to be safe and effective.

## Data availability statement

The original contributions presented in this study are included in the article/supplementary material, further inquiries can be directed to the corresponding author.

## Ethics statement

Ethical review and approval was not required for the study on human participants in accordance with the local legislation and institutional requirements. Written informed consent from the patients/participants or patients/participants legal guardian/next of kin was not required to participate in this study in accordance with the national legislation and the institutional requirements.

## Author contributions

AA and FF organized the database. CP, FF, and DG contributed to the conception and design of the study. CP and AA performed the statistical analysis. IC, GC, RT, CV, and OG contributed to the patients. All authors contributed to the manuscript revision and approved the submitted version.
